# The puzzling inflammatory bowel disease: growing interest for mediators of inflammation

**DOI:** 10.1080/09629359791767

**Published:** 1997-04

**Authors:** F. J. Zijlstra

**Affiliations:** 1 Department of Pharmacology Faculty of Medicine and Health Sciences Erasmus University P.O. Box 1738 Rotterdam 3000 DR The Netherlands


Mediators of Inflammation, 6, 83–84 (1997)

### The puzzling inflammatory bowel disease: growing interest for mediators of inflammation

In this issue of *Mediators of Inflammation* two review articles and one research paper describe the local inflammation in the colon and the role of cytokines, leukocyte migration and the effect of cell–cell interaction on cytokine synthesis. In the paper by Beck and Wallace an overview is given of so far known important pro-inflammatory cytokines (IL-1, IL-2, IL-5, IL-6, IL-8, TNFα and IFNγ) and anti-inflammatory cytokines (IL-4, IL-10, IL-11, IL-13 and TGFβ).[1] In addition the migration of leukocytes, mediated by adhesion molecules such as selectins, chemokines and integrins, is reviewed by van Rees *et al.*[2] During or after this migration lymphocytes could stimulate the synthesis of the pro-inflammatory cytokines IL-2 and IFNγ, as described by Hoang *et al.*[3]

Recently it has been described by Smith *et al.* that IL-1β stimulates the LPS-induced $PGE_2$ synthesis by the colonocyte cell line CACO-2, whereas $LTB_4$ was uneffected.[4] After simultaneous incubation with IL-1ra the elevated levels of $PGE_2$ were completely abolished, although a significant increase in $LTB_4$ was observed. This could be due to the lack of feedback inhibition by substantial amounts of $PGE_2$.

In the December 1995 issue of *Mediators of Inflammation* two debate articles on the involvement of nitric oxide as pro- or anti-inflammatory mediator were published,[5,6] again reflecting Janus, the symbol for mediators of inflammation, which exert both harmful and beneficial functions.

In summary, from the above-mentioned findings it could be concluded that in inflammatory bowel disease (IBD) mediators of inflammation are involved which could be considered as (a) pro- or anti-inflammatory mediators, (b) primary or secondary mediators, (c) proteins, lipid mediators or otherwise, (d) $T_H1$- or $T_H2$-cell derived substances. Furthermore migrated cells could amplify synthesis of mediators by cells localized in the lamina propia. Moreover one should consider that IBD include two different diseases (Crohn's disease and ulcerative colitis) which, though displaying similar aspects of inflammatory features in the colon, nevertheless require distinct medical treatment.

The adequate therapy of patients not responding to classical treatment with corticosteroids nowadays includes the experimental use of anti-TNFα and IL-10 in Crohn's disease,[7,8] whereas nicotine also has proven to be beneficial in ulcerative colitis.[9] Such treatment regimens imply knowledge about mechanisms of action underlying biochemical aspects of acute, subacute and chronic phenomena of colonic inflammation.

So far we may conclude that corticosteroids will suppress all mediators, whereas 5-aminosalicylic acid (5-ASA) partly attenuates cytokine synthesis. Although nicotine probably acts through inhibition of eicosanoids and pro-inflammatory cytokines,[10,11] it is not yet clear why nicotine (including the otherwise harmful habit of smoking) is good for ulcerative colitis and bad for Crohn's disease.[12] This could be related to the $T_H1$- and $T_H2$-cell specific aspects seen in IBD.[13] On the other hand conflicting results were obtained from investigations in Crohn's disease and ulcerative colitis in which IFNγ, IL-2 and IL-10 were all increased.[14,15]

Up to now it is not quite clear whether nicotine selectively inhibits pro-inflammatory cytokines derived from $T_H1$-cells or not, since we observed that IL-10 was decreased after nicotine *in vivo*.[13]

Finally cyclosporin has been reported to be effective in IBD. Both T cell proliferation and the production of T cell-derived cytokines is inhibited.[16] The reduction of T cell migration might be another fruitful area for future studies.[2,17]

Knowledge of the potential T-cell subsets to

© 1997 Rapid Science Publishers generate pro- and anti-inflammatory cytokines in IBD, which in turn affect secondary mediators such as eicosanoids, could contribute to the understanding of these puzzling diseases and subsequently lead to the development of more adequate medicines to prevent severe exacerbations during the chronic phase of the disease.

Submission of research papers on this subject would be welcomed for forthcoming issues of *Mediators of Inflammation*.

*Dr F. J. Zijlstra*
*Assistant Editor*
*Department of Pharmacology,*
*Faculty of Medicine and Health Sciences,*
*Erasmus University, P.O. Box 1738,*
*3000 DR Rotterdam, The Netherlands*
*Fax: (+33) 10 436 6839;*
*Email: zijlstra@farma.fgg.eur.nl*

### References

1. Beck PL, Wallace JL. Cytokines in inflammatory bowel disease. *Mediators of Inflammation* 1997; **6**: 95–103.
2. van Rees EP, Palmen MJHJ, van de Goot FRW, Macher BA, Dieleman LA. Leukocyte migration in experimental inflammatory bowel disease. *Mediators of Inflammation* 1997; **6**: 85–93.
3. Hoang P, Dehennin JP, Li Li, Sibille C, Geubel A, Vaerman JP. Human colonic intraepithelial lymphocytes regulate the cytokines produced by lamina propria mononuclear cells. *Mediators of Inflammation* 1997; **6**: 105–109.
4. Smith GS, Rieckenberg C, Longo WE, Kaminski DL, Mazuski JE, Deshpande Y, Miller TA. The effect of an interleukin receptor antagonist (IL-1ra) on colonocyte eicosanoid release. *Mediators of Inflammation* 1996; **5**: 449–452.
5. Miller MJS, Grisham MB. Nitric oxide as a mediator of inflammation?—You better believe it. *Mediators of Inflammation* 1995; **4**: 387–396.
6. Kubes P, Wallace JL. Nitric oxide as a mediator of gastrointestinal mucosal injury?—Say it ain't so. *Mediators of Inflammation* 1995; **4**: 396–405.
7. Derkx B, Taminiau J, Radema S, Stronkhorst A, Wortel C, Tytgat G, van Deventer S. Tumour necrosis factor antibody treatment in Crohn's disease. *Lancet* 1993; **342**: 173–174.
8. De Vries JE. Immunosuppressive and anti-inflammatory properties of interleukin 10. *Ann Med* 1995; **27**: 537–541.
9. Pullan RD, Rhodes J, Ganesh S, Mani V, Morris JS, Williams GT, Newcombe RG, Russel AH, Feyerabend C, Thomas GAO, Sawe U. Transdermal nicotine for active ulcerative colitis. *N Engl J Med* 1994; **330**: 811–815.
10. Zijlstra FJ, Scrivastava ED, Rhodes M, van Dijk APM, Fogg F, Samson HJ, Copeman M, Russel MAH, Feyerabend C, Williams GT, Pullan RD, Thomas GAO, van Blankenstein M, Wilson JHP, Allen A, Rhodes J. Effect of nicotine on rectal mucus and mucosal eicosanoids. *Gut* 1994; **35**: 247–251.
11. Van Dijk APM, Madretsma GS, Keuskamp ZJ, Zijlstra FJ. Nicotine inhibits cytokine synthesis by mouse colonic mucosa. *Eur J Pharmacol* 1995; **278**: R1–R2.
12. Rhodes J, Thomas GAO. Smoking: good or bad for inflammatory bowel disease? *Gastroenterology* 1994; **106**: 807–810.
13. Madretsma GS, Wolters LMM, van Dijk APM, Tak CJAM, Feyerabend C, Wilson JHP, Zijlstra FJ. In vivo effect of nicotine on cytokine production by human non-adherent mononuclear cells. *Eur J Gastroenterol Hepatol* 1996; **8**: 1017–1020.
14. Kucharzik T, Stoll R, Lugering N, Domschke K. Circulating anti-inflammatory cytokine IL-10 in patients with inflammatory bowel disease (IBD). *Clin Exp Immunol* 1995; **100**: 452–456.
15. Niessner M, Volk BA. Altered Th1/Th2 cytokine profiles in the intestinal mucosa of patients with inflammatory bowel disease as assessed by quantitative reversed transcribed polymerase chain reaction (PT-PCR). *Clin Exp Immmunol* 1995; **101**: 428–435.
16. Faulds P, Goa KL, Benfield P. Cyclosporin: a review of its pharmacodynamic and pharmacokinetic properties, and therapeutic use in immunoregulatory disorders. *Drugs* 1993; **45**: 953–1040.
17. Elson CO, Beagley KW, Sharmanov AT, Fujihashi K, Kiyono H, Tennyson GS, Cong Y, Black CA, Ridwan BW, McGhee JR. Hapten-induced model of murine inflammatory bowel disease: mucosal immune responses and protection by tolerance. *J Immunol* 1996; **157**: 2174–2185.
